# Experiencing an art education program through immersive virtual reality or iPad: Examining the mediating effects of sense of presence and extraneous cognitive load on enjoyment, attention, and retention

**DOI:** 10.3389/fpsyg.2022.957037

**Published:** 2022-09-15

**Authors:** Qingyang Tang, Yanyun Wang, Hao Liu, Qian Liu, Shen Jiang

**Affiliations:** ^1^Cultural Heritage Innovation Lab, School of Journalism and Communication, Beijing Normal University, Beijing, China; ^2^Institute of Communications Research, College of Media, University of Illinois Urbana-Champaign, Champaign, IL, United States

**Keywords:** technological affordance, system immersion, sense of presence, artistic communication, cognitive load, immersive virtual reality (IVR)

## Abstract

Sense of presence and extraneous cognitive load (ECL) are the two psychological effects widely employed to explain the cognitive outcomes caused by high-immersive media (e. g., virtual reality). This study identified the concepts of both technological affordance (i.e., immersion) and the psychological effects of VR learning. It investigated the mechanism by which immersion leads to better or worse communication in the context of art education. We operationalized the concept of immersion into two levels: a high-immersive VR system (HTC VIVE Cosmos) and a low-immersive tablet system (iPad). Through a between-subject experiment, we found that higher immersion not only led to a greater sense of presence but also lowered extraneous cognitive load. Enjoyment and attention increased as a sense of presence rose but were not necessarily predicted by extraneous cognitive load. This study found that sense of presence was a more robust explanatory variable than ECL and that cognitive load could be lower in a high-immersive environment with content specifically designed for VR.

## Introduction

Immersive media plays an increasingly vital role in the field of multimedia learning. Some researchers claimed that immersive virtual reality (IVR) has unique advantages in promoting learning outcomes in terms of learning motivation, enjoyment, interest, knowledge retention, and skill transfer (Parong and Mayer, 2018; Meyer et al., 2019; Zinchenko et al., 2020; Makransky and Mayer, 2022), while others found that IVR is inferior to traditional media in teaching or instructing because of its stressful learning environment or unnecessary cognitive load (Richards and Taylor, 2015; Makransky et al., 2021; Tang et al., 2021). Scholars are also concerned about the extent to which and by what mechanisms new media technologies such as IVR can enhance user experience, achieve the goal of persuasion, and assist receivers' recall (Moro et al., 2019; Liu et al., 2020; Thees et al., 2020; Wang and Yao, 2020; Breves, 2021).

Different theoretical frameworks have been used to explain the benefits and drawbacks of using IVR as the technology to process information or reproduce reality. More specifically, the hypothesis with presence as the explanatory variable is based on the heuristic—a systematic model of information processing that considers that technological affordance (e.g., immersion) is processed as peripheral cues that can trigger some psychological experience (e.g., presence) and has positive effects (Petty and Cacioppo, 1986; Sundar, 2008). The hypothesis with the cognitive load as the explanatory variable is based on the cognitive theory of multimedia learning (CTML; Mayer, 2002), which posits that affordance causes deep processing and that extraneous load has a negative effect on the process (Sweller, 2010). These two lines of research findings seem to indicate that joyful immersive experiences and good recall and retention are hard to achieve at the same time in the IVR environment. Moreover, few studies compared the two perspectives or explored which one provides a better explanation for the mixed results of IVR effectiveness.

This study aimed to 1) explore the effectiveness of IVR in the context of art learning and 2) examine the underlying mechanism and compare the competing explanatory paths (i.e., sense of presence and extraneous cognitive load). A between-subject experiment was carried out to examine the effects of immersion on enjoyment, attention allocation, and retention, as well as the mediating effects of sense of presence and extraneous cognitive load (ECL).

## Literature review

### From immersion to presence: Definition, relationship, and strengths

#### Difference between immersion and presence

The concept of presence was introduced to the field of VR in the 1990s (Lombard and Ditton, 1997; Slater and Wilbur, 1997). At the time, VR technology had just begun to be applied to psychological therapy. Scholars then were interested in the concept of “presence” because they thought that it could help people understand why VR technology is effective in psychological therapy practice, which referred to “the effect VR has on the human psyche” (Schuemie et al., 2001, p. 183). Presence is not exclusively associated with VR. However, VR induces presence through multiple sensory inputs (Dinh et al., 1999; Van Kerrebroeck et al., 2017) and can therefore elicit a more compelling sense of presence than other media forms (Rupp et al., 2016; Van Damme et al., 2019).

Researchers distinguished different terminologies for presence, particularly the crucial concept of immersion and presence. Slater (1999) believed that immersion refers to the objective description of aspects of the system, such as field of view, image latency, and frame rate of the image stream, while presence refers to a subjective phenomenon, such as the sensation of being in a virtual environment (VE). Witmer and Singer (1998) stated that presence was a normal awareness phenomenon that required directed attention. These early studies emphasize the fundamental distinction between presence and immersion; presence relates to the user's subjective experience, whereas immersion refers to the objective feature of immersive technology.

In more recent studies, researchers defined presence in more specific ways. Shin (2019b) and Teng (2010) described immersion as becoming part of the experience itself, physically or virtually. In this case, the definition of immersion is less relevant to the system and more about the human experience. Shin (2019a, p. 304) also argued that presence can be viewed as “a state of mind,” whereas immersion is “an experience in time.” In other studies, presence was treated as a state of consciousness reliant on the perception of “being there” in VR environments and as “a psychological state in which virtual objects are experienced as actual objects in either sensory or nonsensory ways” (Riches et al., 2019; Bermejo-Berros and Gil Martínez, 2021; Cerda et al., 2021). Fromberger also provided some ideas to differentiate the two concepts: immersion “is an objective description of aspects of the technological system and can be increased,” while presence “is a psychological phenomenon and can be defined as the feeling of being in one place or environment even when one is physically situated in another” Fromberger et al. (2015, p. 2).

From the perspective of human–computer interaction (HCI), technological affordance can trigger heuristics through psychological cues to impact users' ways of processing content (Sundar, 2008), and presence is referred to as a typical heuristic caused by immersion affordance (Sundar, 2008; Sundar et al., 2017; Duan et al., 2021). The heuristic is a mental shortcut that provides an effortless way for users to assess the quality of the message and invisibly affects the motivation needed to take in and remember information (Chaiken, 1980; Petty and Cacioppo, 1986). Provided by the immersive perceptual interface, a sense of presence could trigger the being-there heuristic, thus leading to a positive attitude and then selective retention of the content (Sundar, 2008; Sundar et al., 2017). Presence has long been used to explain the communication effects of immersive media and is known as one of the most direct advantages of immersion (Kim and Biocca, 1997; Lombard and Ditton, 1997; Wirth et al., 2007; Breves, 2021).

To summarize, most scholars in HCI tend to classify immersion as an attribute of a technical system that is an important factor that triggers presence. However, in a broader context, presence is not exclusively associated with high immersion. It is a psychological status that could be achieved by attributing more mental effort to a less immersive environment. Meanwhile, people can perceive different levels of presence even in the same media environment.

#### How does presence benefit enjoyment and attention?

Enjoyment refers to the degree of positive emotion thatusers feel when interacting with a digital system. Sherry (2004) explains that media enjoyment is realized in a flow state when an individual can interpret the mediated content without difficulty or boredom. Users in a more immersive environment might experience more enjoyment because they feel that they are part of a high-fidelity virtual environment with meaningful social interactions (Makransky and Mayer, 2022). Presence is positively correlated with mood experience, game enjoyment, and satisfaction and gratification, which contribute to an appealing experience (Sylaiou et al., 2010; Ho et al., 2017; Tussyadiah et al., 2018. Lee et al. (2013) further illustrated that presence could significantly predict perceived enjoyment in an IVR context. It is assumed that system immersion benefits perceived enjoyment and positive perceptual experience by prompting greater psychological presence compared to more conventional media (Sundar, 2008; Makransky et al., 2021).

To a certain extent, VR offers users a virtual space and a fully occupied view that reduces the user's perception and attention to physical space. Compared with framed screens, users are less likely to be distracted by information other than media content. The sense of presence has been widely used as the psychological reason why immersion can make people focus. Kim et al. (2021) found that presence could increase attention; however, this effect varied between genders. Wang and Yao (2020) believed that presence led to a reduction in the capacity for processing peripheral information, thus making users focused on the main task. The novelty cue might also be triggered by the perceived presence, especially for individuals who were inexperienced with IVR. With this explanation, IVR could attract more attention from users than other traditional media (Sundar, 2008).

#### Presence and memory

Controversy remains regarding the memory effect (as reflected in knowledge tests of retention, behavioral tests of transfer, etc.) of presence (Makransky et al., 2019b; Meyer et al., 2019; Kim et al., 2021). Some studies believe that presence can make information in a mediated environment more accessible and improve memory of media content (Kim and Biocca, 1997). The more the viewer is present in the mediated environment, the more the amount of information about the mediated environment processed by the viewer is expected to increase (Dinh et al., 1999; Cho, 2018). For example, Dinh et al. (1999) stated that a greater presence increases people's memory of objects in a virtual environment. In contrast, some evidence suggests that the memory effect of presence is feeble (Cadet and Chainay, 2020; Loureiro Krassmann et al., 2020; Morélot et al., 2021). For example, Nelson et al. (2006) found that participants recalled less information about brands in a video game if they perceived a greater level of presence. The high immersion elicits a greater level of presence, which requires more intensive investment in cognitive capacity because the perception and complexity in the high immersive environment includes more sensory information that would occupy cognitive resources (Roettl and Terlutter, 2018). In this case, according to the limited capacity model (Lang, 2000), the high presence may lead to a reduction in the capacity for processing and remembering information.

In conclusion, researchers did not agree on how presence might affect memory, and the empirical evidence pointed to a mixed effect through different processes. This study aims to explore further which mechanism explains the effectiveness more accurately.

### Cognitive overload: The hackneyed disadvantage of immersive media

When researchers discuss why IVR leads to poor learning outcomes, such as less retention, transfer, and more distraction, higher cognitive load or overload—especially the extraneous component—has been frequently mentioned (Richards and Taylor, 2015; Frederiksen et al., 2020; Makransky et al., 2021). The cognitive load model proposed by Sweller (1988) divided the load in limited working memory capacity into three types: intrinsic cognitive load (i.e., “element interactivity,” which depends on how the learning content itself interacts with the learners' prior knowledge), germane cognitive load (i.e., the cognitive resources devoted to schema construction and mental processes storing information into long-term memory), and ECL (ECL, i.e., totally unhelpful load caused by inappropriate instruction design, such as redundant or distracting elements and unclear explanations). Among the three types, ECL was identified as a drawback that may exhaust the cognitive capacity saved by other factors to explain the not better or worse memory result of IVR compared with nonimmersive media (Sweller, 2010; Makransky and Petersen, 2021).

Given the role of ECL in cognitive load theory, we aimed to explore whether it is the cause of poorer cognitive outcomes from immersion in the context of art learning. Specifically, through the experiment, we attempted to clarify the following questions. First, is ECL the overwhelming negative mental effect of immersion? Second, can the assumption of ECL explain the poor performance of memory in the IVR condition compared with low immersive media?

#### Relationships between immersion, cognitive load, and memory

A large number of studies suggested that IVR does not perform as adequately as nonimmersive media (e.g., mobile phones, iPads, laptops, and 2D televisions) in terms of retention (Schrader and Bastiaens, 2012; Frederiksen et al., 2020; Liu et al., 2021) and recall (Shen et al., 2020; Wang and Yao, 2020) of declarative messages (Khot et al., 2013; Birbara et al., 2020). Slobounov et al. (2015) used electroencephalography (EEG) to demonstrate that fully immersive 3D presentations require more brain effort than a 2D environment for motor control. Frederiksen et al. (2020) employed secondary-task reaction time at different phases to reflect cognitive load. They found that IVR training induced higher cognitive load and worse learning performance than 2D screen training. Although these studies did not specify and measure the three subtypes of cognitive load, given the features of VR, such as higher amounts of sensory stimuli, complex visual and audio sources, and excessive information in the panoramic field of view, it is reasonable to infer that high immersion could increase ECL.

Some researchers note that the specific technical performance of immersion, such as higher amounts of sensory stimuli and a more stereoscopic vision of the 3D model, leads to visual–audio complexity and cognitive overload (Frederiksen et al., 2020; Albus et al., 2021; Makransky et al., 2021). In addition, the panoramic field of view makes users need more cognitive resources to locate and find what to focus on (Makransky and Petersen, 2021). Prior evidence showed that IVR tends to induce greater cognitive load and less memory; however, causality has not been demonstrated.

Some competing theories and evidence suggest that high immersion does not always lead to higher ECL (Andersen et al., 2016; Greenwald et al., 2018). ECL is responsible for cognitive outcomes in an immersive environment (Howard and Lee, 2020). Makransky et al. (2019a) used an EEG to measure cognitive load and found no difference between IVR and 2D screens. Whether the IVR introduced cognitive load is influenced by duration, indicating that overload is not an issue if one uses VR for only a short time. According to Andersen et al. (2016), the cognitive load-reducing principle used in the IVR medical technology training scenario resulted in significantly higher cognitive load. A similar conclusion was found by Howard and Lee (2020) that a habituation pretraining intervention for the cognitive load was ineffective in improving short-term test grades for VR training, whereas attentional advice for distraction successfully brought higher retention. Therefore, it could be speculated that the problem with IVR was not cognitive overload but a distraction. Other studies suggest that immersion could reduce ECL by facilitating direct spatial interaction (Regian et al., 1992) and simplifying user interface operation through intuitive head interaction (Greenwald et al., 2018).

According to the studies reviewed thus far, the experimental data and assumptions are rather controversial. There is no general agreement about whether immersion leads to poor memory, especially whether the ECL is responsible for it.

#### The negative impact of ECL on enjoyment and attention

In addition to memory, we also examined the effects of ECL on other aspects: enjoyment and attention allocation. Some studies have demonstrated a negative relationship between external cognitive load, enjoyment (Renninger and Hidi, 2016; Chang et al., 2017; Lin et al., 2019), and attention (Howard and Lee, 2020; Wang and Yao, 2020; Hughes et al., 2021), especially in IVR scenarios.

When people are in a flow state, they enjoy the experience and perceive little cognitive load. The working memory capacity should be fully occupied; otherwise, people will be bored rather than enjoying themselves, which requires an optimal load state (Wosnitza et al., 2009, p. 80). Nevertheless, the ECL caused by a poorly organized content that makes information difficult to understand is clearly negative for enjoyment. For example, Chang et al. (2017) provided evidence that flow experience negatively correlates with ECL (Chang et al., 2017).

Hughes et al. (2021) indicated a significant positive correlation between distraction and ECL. The attention allocated to the main content was reduced by some unrelated cognitive effort. Redundant and seductive details that trigger extraneous loads are considered the typical negative side of IVR (Van Der Heijden, 2004; Howard and Lee, 2020). It is unhelpful to the delivery of target content (i.e., the unknown facts about the Mona Lisa) to take up attention resources (Makransky et al., 2019a).

### Gaps of previous studies and rationale of this study

The inspiration and rationale for the present research come from two limitations of earlier investigations. The first is that measurement and manipulation refinement are insufficient, making presence and ECL self-evident strengths and weaknesses of immersion, respectively, instead of being supported by sufficient evidence (Anmarkrud et al., 2019). Specifically, most research chooses the operation of immersion or self-reported presence separately, without taking presence as a mediator and uncovering the psychological motivation behind the benefits of immersive affordance. As Sundar (2008) pointed out, technological hope does not equal psychological reality. Some studies suffer from taking presence as the explanation of outcomes only with correlation and making an unsound prediction (Kweon et al., 2018; Borbála, 2018; Lin et al., 2019).

The current study, therefore, attempts to provide evidence for the validity of this path using a mediation model test. We selected the presentation media based on their levels of modality and interactivity. According to Sundar et al. (2017), IVR is richer than the iPad. For modality, the head-mounted display can block the interference of the real physical world; a higher visual field enlarges the perceptual bandwidth, allowing a more natural interaction, such as moving one's head instead of fingers to navigate the virtual world (Sundar et al., 2017).

The measurement and attribution of cognitive load are rather complicated. This is partly because the relevant theories in educational psychology are evolving in response to the challenges outlined in the literature reviews mentioned above. Specifically, some studies tested cognitive load but were unable to identify the different effects of the three load types in the case where GCL and mental effort may be beneficial to learning at an appropriate level (Slobounov et al., 2015; Andersen et al., 2016; Roettl and Terlutter, 2018; Makransky et al., 2019a). We also noticed that the negative effect of IVR compared with other nonimmersive media was attributable to an increase in cognitive load, but no direct measure of the cognitive load was conducted to confirm the statement (Richards and Taylor, 2015; Wang and Yao, 2020; Liu et al., 2021). Hence, the present research aimed to use self-reporting to test the specific extraneous load not conducive to optimizing the user experience, attracting user attention or enhancing content recall.

Another problem is that most previous studies did not consider the impact of content type, fineness, and device details. Some studies have not categorized the viewing materials and devices rigorously. For example, the information delivered by immersive (e.g., HMDVR, 3D television) and 2D media (e.g., text, PPT, picture, and screenshot) are different in the first place. The difference between VR and graphic learning materials is not only their immersive nature. It is also possible that the recording of VR content may lose some information in the side or back view. Some content of low quality (e.g., filming issues or edition from the PC version) may cause high cognitive load, low presence, or other poor cognitive outcomes due to inappropriate content organization, which should not be blamed on the technological affordance of immersion per se (Makransky et al., 2019a).

Based on prior research, we selected the material called the Mona Lisa: Beyond the Glass. This content is for art education, which has received less attention in the field of multimedia learning (Hamilton et al., 2021). It provides knowledge about the world's most famous paintings from the perspective of the author's life, background story, painting techniques, and art appreciation. It also provides a more intimate way for users to appreciate the paintings. Users on the content distribution site have given the content positive feedback, such as “good knowledge dissemination content” and “the production shows off the VR features.” We anticipated that this VR production would be of high quality and could create a different experimental effect.

**Figure 1:**
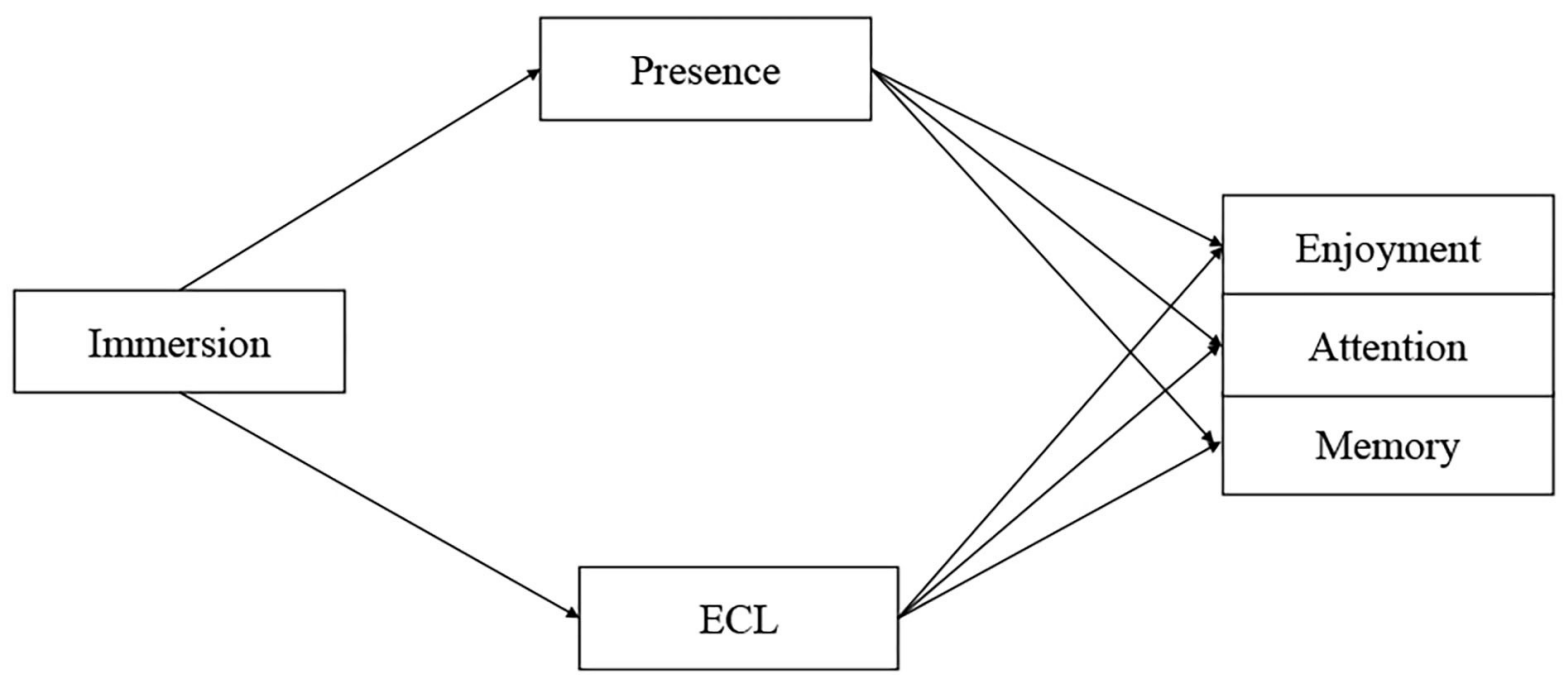
Hypothetical model.

To conclude, we found a convergence trend between HCI and multimedia learning in the challenges of interactive, immersive, and intelligent media as learning or persuasive tools. As stated above, there are competing arguments and inconsistent evidence regarding the psychological effects and cognitive outcomes of immersion (see Figure 1). Therefore, we raised the following research questions to examine the effects of immersion on presence and ECL and how the presence and ECL influence learning outcomes (enjoyment, attention allocation, and retention), as well as the mediating effects of sense of presence and ECL.

RQ1: Does immersion necessarily increase both presence and ECL?
RQ2: How do presence and ECL influence the enjoyment, attention allocation, and knowledge retention of content?
RQ3: Which indirect route can better predict the outcome variables, immersion–presence–benefit or immersion–extraneous load–downside?

##  Method

A between-subject randomized experiment was conducted in Beijing, China. We manipulated the concept of immersion into two levels: a high-immersive VR system and a low-immersive iPad system.

###  Participants

Sixty participants in the study were recruited from a public university in Beijing through online posters. Eventually, there were 29 female and 30 male participants who qualified. Their ages ranged from 17 to 26 years old (M = 20.07, SD = 2.132). They were randomly assigned to two conditions, with 29 in the low-immersion group and 30 in the high-immersion group.

###  Materials and apparatus

An art VR program called the Mona Lisa: Beyond the Glass, jointly developed by Le Louvre and HTC VIVE, was adopted as the content material (downloaded from https://store.steampowered.com/app/1172310/Mona_Lisa_Beyond_The_Glass/). This VR content can be played on three types of media, including HMD VR, mobile VR, and iPad versions, enabling researchers to present the same content through different media techniques. The device used by the high-immersion group was VIVE Cosmos, which supports a wide field of view (FOV) and six degrees of freedom (6DoF); the low-immersion group used iPad Pro 2018, with which participants could view content by swiping the screen or turning the device.

###  Procedure

Before the experiment, the participants completed an online questionnaire focusing on their knowledge of the Mona Lisa painting, how much they liked it, whether they had seen it in person at the Louvre, and some demographic information. After signing the informed consent form, the experimenter helped the participants set up the devices and explained how to experience the program through an iPad or HMD VR.

Each participant entered the experiment room and finished the content alone without any interruption. The viewing experience lasted approximately 10 mins. After that, the participants were asked to complete a post-test questionnaire. Finally, all participants received 20 RMB (approximately $3.5) as a reward for participation.

### Measures

*Outcome variables*: The participants were asked to report whether they had enjoyed the viewing experience, the viewing method, and the viewing content by answering three questions adopted from previous research (Sundar et al., 2017; Liu et al., 2020). Attention allocation was measured using the questions adopted from the same research by Sundar et al. (2017) (e.g., “I devoted my whole attention to the story”). Retention was assessed *via* a knowledge multiple-choice (MC) questionnaire developed according to the stimuli (eight items, see Appendix 1). Considering that the content of this video mainly entails an introduction and some facts about the world-famous painting, we used MC to test the retention of basic facts as a reflection of memory level. The total number of correctly answered questions was summed for analyses.

*Mediators:* Sense of presence was measured using the scale from Kim and Biocca (1997) (e.g., “The content I experienced came to me and created a new world for me, and the world suddenly disappeared when the viewing ended”). We adopted the measurement from Leppink et al. (2013) to measure ECL (e.g., “The instructions and/or explanations during the activity were very unclear”).

*Control variables:* Past research suggests that simulator sickness, depending on personal condition, is relevant when predicting the effect of the VR experience (Bailenson, 2018; Birbara et al., 2020). The simulator sickness questionnaire (SSQ) developed by Kennedy et al. (1993) was also included in the posttest. We also controlled other demographics (i.e., gender and age) and baseline levels of prior knowledge (“How much do you know about the painting *Mona Lisa*”; 1 = not at all, 5 = very much) together with likeness (“How much do you like the painting *Mona Lisa*”; 1 = not at all, 5 = very much).

## Results

### Different outcomes between the two levels of the immersive group

The results of independent sample *t*-tests showed significant main effects of immersion on presence [*t* (57) = −3.325, *p* = 0.002] and extraneous load [*t* (57) = 3.034, *p* = 0.004]. Viewing the content in a high-immersion environment (M = 3.442, SD = 0.840) elicited a higher level of presence than viewing on an iPad (M = 2.746, SD = 0.764), together with a lower level of extraneous load (M = 1.389, SD = 0.411) than viewing the content in the low-immersion group (M = 1.874, SD = 0.769). Therefore, the answer to RQ1 in this study is that immersion increases presence but simultaneously decreases ECL. Enjoyment [*t* (57) = −3.627, *p* = 0.001] and attention allocation [*t* (57) = −2.863, *p* = 0.006] also showed significant differences between the two groups, with the high-immersion group reporting greater enjoyment (M = 4.433, SD = 0.662) and higher attention (M = 4.108, SD = 0.798) than the low-immersion group (M_enjoyment_ = 3.804, SD = 0.670; M_attention_ = 3.491, SD = 0.857). However, no difference in retention [*t* (57) = 1.060, *p* = 0.294] was observed between the two groups (M_high_ = 6.23, SD = 1.431; M_low_ = 6.62, SD = 1.374).

### Mediation effects of presence and extraneous cognitive load

**Figure 2:**
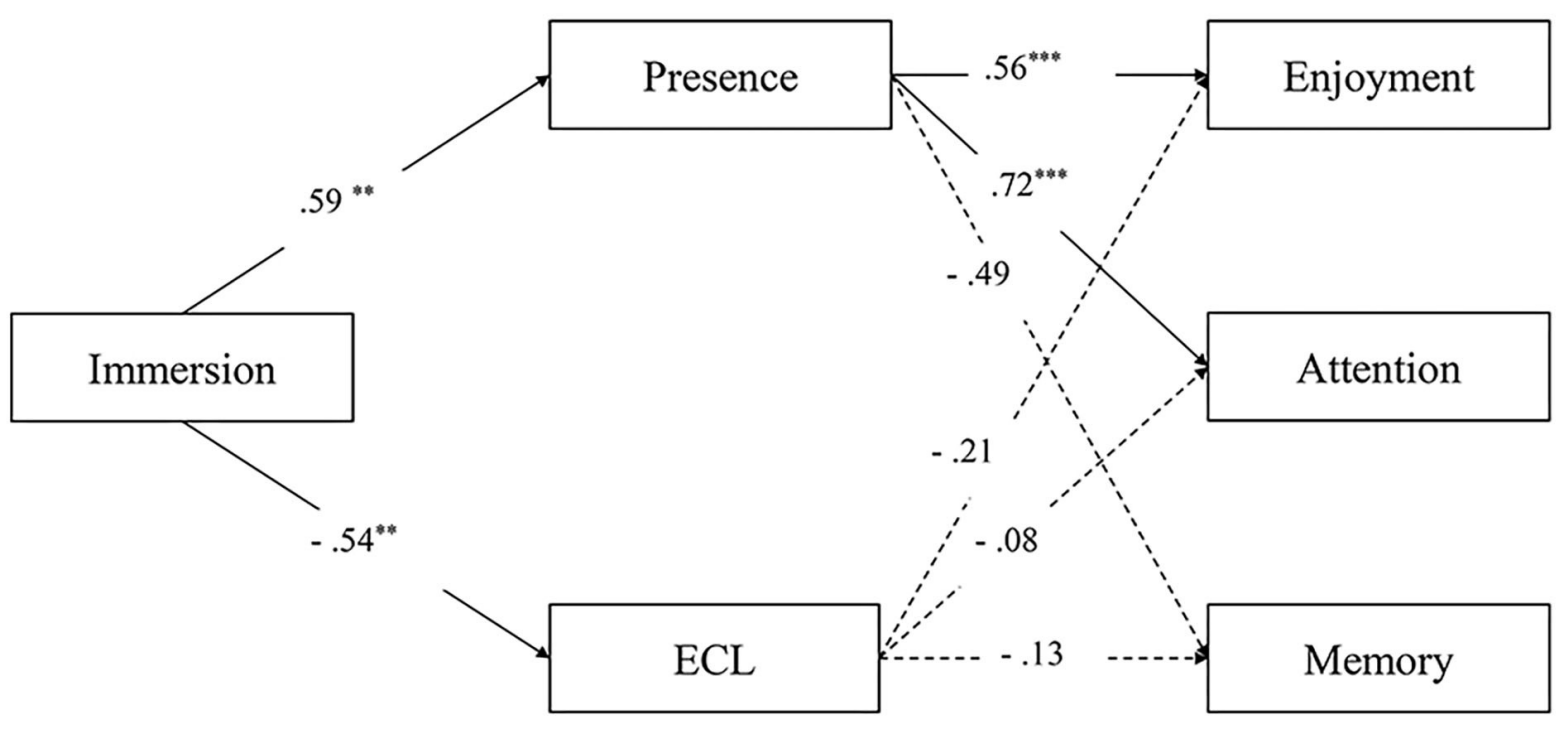
Mediation models. * = *p* < .05. ** = *p* < .01. *** = *p* < .001.

To address RQ2 and 3, we conducted a mediating analysis to test the mechanism of how immersion works on the human psyche *via* presence and cognitive load (see Figure 2). Mediation models were tested with PROCESS Macro v3.5 (95% CIs, 5,000 bootstrap samples; Hayes, 2018). Gender, age, prior knowledge, likeness, and SSQ were entered as covariates. We used Model 4 to test the mediation effects with enjoyment, attention allocation, and knowledge retention as dependent variables separately, immersion as an independent variable (0 = low, 1 = high), and presence and ECL as mediators.

The results showed that immersion had a significant positive impact on presence (b = 0.59, se = 0.20, t = 3.01, *p* = 0.004) while demonstrating a significant negative impact on extraneous load (b = −0.54, se = 0.17, t = −3.08, *p* = 0.003); thus, the first step was supported. Then, we found that presence elicited enjoyment (b = 0.56, se = 0.10, t = 5.8, *p* = 0.0001) and attention allocation (b = 0.72, se = 0.12, t = 5.94, *p* = 0.0001). Immersion's indirect effect on enjoyment (a^*^b = 0.33, BootSE = 0.14) and attention allocation (a^*^b = 0.42, BootSE = 0.16) *via* presence was significant. However, no impact of ECL on enjoyment (b = −0.21, se = 0.11, t = −1.89, *p* = 0.064) or attention (b = −0.08, se = 0.14, t = −0.601, *p* = 0.550) was found. In addition, no impact of presence (b = −0.49, se = 0.26, t = −1.86, *p* = 0.068) or extraneous load (b = −0.13, se = 0.29, t = −0.46, *p* = 0.650) on retention was demonstrated in the presented experiment. These results responded to RQ2.

Overall, regarding RQ3, we found that the influence of immersion on enjoyment and attention allocation was mediated by the subjective feeling of presence, while ECL did not stand out as a significant mediator between immersion and any outcomes. The immersion–presence–benefit route better predicted outcome variables and explained the mechanism of immersion eliciting the human psyche.

## Discussion

In the current study, we explored presence and ECL caused by different levels of immersion and how these two psychological factors influence the dissemination and educational effects of artwork: enjoyment, attention allocation, and knowledge retention. In particular, we attempted to compare the competitive explanatory mechanism and determine which factor could better explain and predict learning effectiveness. As our study results revealed, a sense of presence had a higher explanatory power on the influence mechanism of immersion than ECL when the virtual representation of artwork was appropriately designed.

Specifically, our results revealed that participants perceived more presence in the high-immersion condition, which is consistent with previous research findings that a higher level of system immersion would bring a greater sense of presence (Shen et al., 2020; Makransky et al., 2021). Interestingly, we found that ECL was lower in the IVR group than in the 2D group. This finding contradicts other findings under a game-based learning scenario that IVR causes higher ECL as a cost of enjoyment and motivation (e.g., Kweon et al., 2018). A possible explanation for our finding could be that our material was designed specifically for IVR instead of being adapted from an existing 2D video, which maximized the advantages of IVR features and was appropriate for IVR presentation (Baceviciute et al., 2020). This result indicated whether the content was suitable for presentation in IVR did matter (Howard et al., 2021) and whether ECL was not necessarily positively related to the immersion level of media, as previous research suggested (Khot et al., 2013; Birbara et al., 2020; Liu et al., 2021; Makransky and Petersen, 2021).

We did not find a significant relationship between retention and any of the predictors (i.e., immersion, presence, and ECL). As we measured immediate retention as a reflection of memory level, our results suggested that, when the message in the viewing material was the same, the information conveyed would not be impacted by the media platforms. This is consistent with the finding of Makransky et al. (2019b), which suggests that IVR, 2D desktop, and text are equivalent in delivering basic knowledge. Another possible explanation is that memory is not affected by technical affordance when it comes to immersion and is weakly related to psychological effects such as presence and ECL in the field of art education. Instead, we found that simulator sickness significantly negatively affected retention after all other variables were controlled (b = −1.669, t = −2.318, *p* = 0.025). Hence, similar to Birbara et al. (2020) suggestion, we proposed that physical simulator sickness could be a major factor in the immersive learning environment instead of mental effort.

This research also adds to the body of literature on the mechanism of how presence and ECL explain the relationship between immersion and final cognitive effects. We tested multiple mediator models and found a mediation effect of presence between immersion and enjoyment. Presence is also the mediator between immersion and attention. However, no significant mediation effect was found for ECL, and neither of these two potential mediators could predict the memory of factual knowledge about the Mona Lisa. Our findings draw more parallels to the immersion–presence–benefit route and fewer parallels to the immersion–extraneous load–downside route. This suggests that the sense of presence can explain more in our case, while ECL does not have to be a downside of high immersion.

Our study has practical implications for IVR content production. With the development of interactive VR content (Gunkel et al., 2017; Ozacar et al., 2017; Reyes and Zampolli, 2017; Wilson and McGill, 2018), the content was designed independent of IVR, and highlighting the immersive features of IVR has become accessible. In recent years, a new technical environment has made VR studies different from those in the past. Our research introduced the Mona Lisa: Beyond the Glass as the viewing material and found that, when played in IVR, it can produce less ECL than in a low-immersion 2D display. We suggest the possibility of immersive affordance saving cognitive resources if the content is designed appropriately.

Although it did not cause better memory outcomes, we believed that immersion could still represent the advantage of IVR over 2D displays. It is effective to use high-immersion technologies to display artworks when local museums are not accessible. Unlike formal education, in a context such as artistic literacy, cultivation, attitude, enjoyment, and attention matter more because they motivate people to learn about nonutilitarian knowledge. The current research also adds evidence to the application of immersive learning in art education, which previously has not received adequate attention from scholars (Hamilton et al., 2021).

Additionally, the strategy used in the content design of the *Mona Lisa: Beyond the Glass*, an excellent VR exhibition of the world-famous painting, combined with our empirical results, can add some practical experience to the VR industry. It employed “fade to black” to guide the audience's attention to the content creators' point of interest, which has been confirmed to be effective (Danieau et al., 2017). Specifically, only the Mona Lisa's avatar and related iconography seem illuminated. The side and back areas are dark, using clean lines to outline a sense of space, but without too many meaningful elements to distract attention. In addition to graphic design, the natural method of interaction, as an essential aspect of immersion, also has a significant impact on the cognitive load.

Our findings of the VR material supported the principles found in other studies that could be used in art education or VR museums. For instance, Greenwald et al. (2018) proposed that a 2D screen with a stylus was less intuitive and required instruction, and using the hands instead of the head probably caused ECL. As Kiyokawa (2008) suggested, using low-immersive media to watch 360-degree content could also result in missing the information collected from the peripheral view in the high-immersive environment due to the lack of field of view; thus, an IVR display should be provided to elicit less ELC.

There are several limitations to this study. First, self-reported measurement of ECL only allows comparison of size differences between groups but it does not reflect whether the user is overloaded, which could affect the interpretation of the results (Leppink et al., 2015). The combination of the scale and the EEG-based real-time cognitive load test provides a better presentation of cognitive load, which should be considered in the future (Gerry et al., 2018). Second, this experiment is short-term. Memory is also reflected by transient retention, which may be more likely to stimulate peripheral processing rather than the deep processing required for grasping knowledge. Future research should test long-term memory effects to explore whether viewers benefit from presence through embodied cognition to have better memory outcomes. Third, the instructional material used in this study was a 10-min VR experience rather than continuous and structured lessons; thus, we measured retention in only eight questions. Although these items already covered the knowledge points in this VR content, the number of items being too low to control many aspects of errors or reflect accurate memory levels is also a limitation due to the lack of complexity of the experimental material. The learning content and test were of low difficulty and did not cause overload, even with ECL, which could also be why cognitive load probably did not substantially impact memory. In the future, we can develop the measurement of overload and increase the difficulty of the task. In addition, we used a between-subject design in the current study, which is probably not the most proficient design to test a mediating model. As the connection between system immersion and psychological presence was validated by our and others' work, future research could pay more attention to the psychological process of how presence influenced participants' learning outcomes.

##  Conclusion

In the present study, we mainly found that immersion could lead to a higher presence and, thus, higher cognitive outcomes in IVR but did not result in higher ECL. The association between ECL and cognitive outcomes was insignificant, suggesting that ECL may not be the reason for the poorer message delivery of immersion. Instead, other factors may be more relevant. For example, as a control variable in this study, SSQ was found to impact retention negatively. The development of IVR exclusive content has caused different results from previous predictions, mainly in ECL. VR technology may be more suitable for mass communication application scenarios with entertainment-based soft knowledge popularization than nonimmersive media. In addition, our study was an initial attempt to integrate explanations of VR effects from different fields and provides empirical evidence. We suggest that VR scholars from different fields should understand theories in other disciplines and that we need more collaboration to work together to understand how VR affects human perceptions and behaviors.

## Data availability statement

The original contributions presented in the study are included in the article/Supplementary material, further inquiries can be directed to the corresponding author/s.

## Ethics statement

The studies involving human participants were reviewed and approved by the Ethic Committee of School of Journalism and Communication, Beijing Normal University. Written informed consent for participation was not required for this study in accordance with the national legislation and the institutional requirements.

## Author contributions

QT designed the study, wrote the draft, and contributed most to the study. YW and HL participated in the draft writing and data collection. QL conducted the data analysis and revised the manuscript. SJ supervised the research project and revised the manuscript. All authors participated in the writing and revisions of the manuscript.

## Funding

This study was supported by the Beijing Social Science Fund (grant no. 19XCC015).

## Conflict of interest

The authors declare that the research was conducted in the absence of any commercial or financial relationships that could be construed as a potential conflict of interest.

## Publisher's note

All claims expressed in this article are solely those of the authors and do not necessarily represent those of their affiliated organizations, or those of the publisher, the editors and the reviewers. Any product that may be evaluated in this article, or claim that may be made by its manufacturer, is not guaranteed or endorsed by the publisher.
