# Supplementary material for: Experiencing an art education program through immersive virtual reality or iPad: Examining the mediating effects of sense of presence and extraneous cognitive load on enjoyment, attention, and retention
Source: Front Psychol. 2022 Sep 15;13:957037. doi: 10.3389/fpsyg.2022.957037 (PMC9521546; doi:10.3389/fpsyg.2022.957037)
Supplement: Supplementary file 1 [file Table_1.DOCX]

Supplementary Material

# Appendix

1 The painting "Mona Lisa" was painted by Leonardo da Vinci from which year

A 1501

B 1502

C 1503

D 1504

2 The painting Mona Lisa was hung in a bathroom by which king and then deformed by moisture causing cracks

A François I

B Charles V

C Louis XII

D Henry II

3 On which material was the Mona Lisa painted?

A oil on canvas

B wood on board

C stone on board

D paper on board

4 How old was Mona Lisa when she started painting?

A 10-20 years old

B 20-30 years old

C 30-40 years old

D 40-50 years old

5 Was the model of the Mona Lisa pregnant at the time of the painting?

A Yes

B No

6 Which of the following statements about the archetypal figure of the Mona Lisa is true.

A She was called Lisa Dell Giocondo, wife of the wealthy Florentine fabric merchant Francesco, and was known as Gioconda

B She was called Lisa Dell Giocondo, wife of the wealthy Florentine fabric merchant Francesco, and she was known as the Mona Lisa

C The Mona Lisa was painted spontaneously by Leonardo da Vinci when he saw the particular beauty of the archetypal figure, and was not custom-made by the owner

D The painting Mona Lisa was custom-made by the couple of the archetypal figure to celebrate the fact that Mona Lisa was pregnant

7 Painters sometimes leave an element on a portrait to suggest the identity of the subject, which is the most important special element in the Mona Lisa that suggests identity

A The prickly cypress branches of Mona Lisa's audience

B Mona Lisa's folded hands

C The smiling expression of the Mona Lisa

D Mona Lisa's floppy hat

8 Which of the painting methods mentioned in the material just viewed created the Mona Lisa's enigmatic smile

A semi-thick paint B halo paint C vignetting D stippling

9 Which of the following statements is false

A The landscape behind the Mona Lisa is fictional and the result of the artist's constant observation of nature

B The landscape behind the Mona Lisa uses the principle of air perspective

C The landscape behind the Mona Lisa is real
